# The Small RNA Landscape in NSCLC: Current Therapeutic Applications and Progresses

**DOI:** 10.3390/ijms24076121

**Published:** 2023-03-24

**Authors:** Giuseppe Ciccone, Maria Luigia Ibba, Gabriele Coppola, Silvia Catuogno, Carla Lucia Esposito

**Affiliations:** 1 Institute of Experimental Endocrinology and Oncology “Gaetano Salvatore” (IEOS), National Research Council (CNR), 80145 Naples, Italy; 2 Department of Environmental, Biological and Pharmaceutical Sciences and Technologies, University of Campania “Luigi Vanvitelli”, 81100 Caserta, Italy; 3 Department of Precision Medicine, University of Campania “Luigi Vanvitelli”, 80138 Caserta, Italy

**Keywords:** NSCLC, aptamer, ASO, RNAi, targeted therapy

## Abstract

Non-small-cell lung cancer (NSCLC) is the second most diagnosed type of malignancy and the first cause of cancer death worldwide. Despite recent advances, the treatment of choice for NSCLC patients remains to be chemotherapy, often showing very limited effectiveness with the frequent occurrence of drug-resistant phenotype and the lack of selectivity for tumor cells. Therefore, new effective and targeted therapeutics are needed. In this context, short RNA-based therapeutics, including Antisense Oligonucleotides (ASOs), microRNAs (miRNAs), short interfering (siRNA) and aptamers, represent a promising class of molecules. ASOs, miRNAs and siRNAs act by targeting and inhibiting specific mRNAs, thus showing an improved specificity compared to traditional anti-cancer drugs. Nucleic acid aptamers target and inhibit specific cancer-associated proteins, such as “nucleic acid antibodies”. Aptamers are also able of receptor-mediated cell internalization, and therefore, they can be used as carriers of secondary agents giving the possibility of producing very highly specific and effective therapeutics. This review provides an overview of the proposed applications of small RNAs for NSCLC treatment, highlighting their advantageous features and recent advancements in the field.

## 1. Introduction

Non-small-cell lung cancer (NSCLC) represents the majority of LC covering about 80–85% of all cases. It includes adenocarcinoma, squamous cell carcinoma and large-cell carcinoma subtypes. NSCLC is the most common cancer worldwide and a leading cause of cancer death, with more than 1.6 million deaths each year, according to the World Health Organization [1].

The main therapeutic approaches for NSCLC treatment include surgery, chemotherapy with platinum-based compounds and/or radiotherapy. In addition, for some subgroups of patients with advanced NSCLC, targeted therapies with Tyrosine Kinase inhibitors (TKIs) and immunotherapies with anti-Programmed Death-Ligand 1 (PD–L1) monoclonal antibodies (mAbs) are frequently used. Despite advances, the 5-year relative survival rate for NSCLC patients is about 26%, and the resistance to therapies and relapses are very recurrent [2]. Therefore, the development of new targeted therapies and strategies for restoring patients’ responsiveness to common drugs is essential.

**Figure 1:**
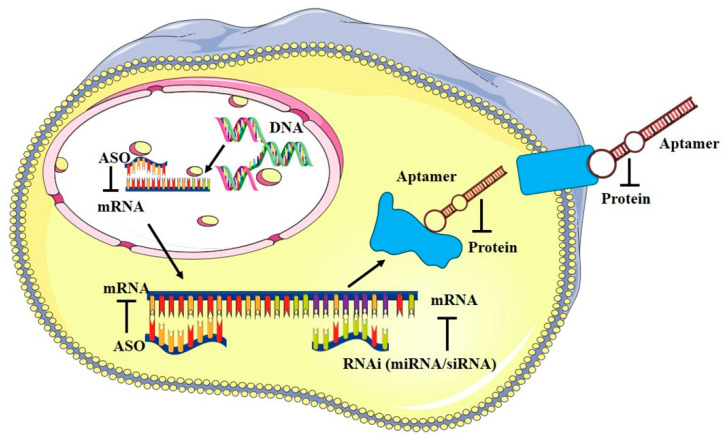
RNA-based therapeutics general mechanism of action. Scheme of the main mechanism of action of small RNA-based therapeutics: ASOs, miRNAs, and siRNAs bind to mRNA preventing proteins translation; aptamers bind their target protein by folding into complex tridimensional shapes.

In this context, there is a growing interest in small RNA-based therapeutics including Antisense Oligonucleotides (ASOs), microRNAs (miRNAs or miRs), short interfering RNAs (siRNAs) and aptamers. ASOs, miRNAs and siRNAs act by targeting and inhibiting specific target mRNAs, whereas aptamers are 3D-structured oligonucleotides serving as high-affinity ligands and potential antagonists of disease-associated proteins (Figure 1).

In addition, more recently, small double-strand RNAs (dsRNAs), able to activate endogenous genes by a mechanism of a gene promoter targeting involving epigenetic modifications, have been described and termed small activating RNAs (saRNAs). This new class of small RNAs shows great potential; however, their application to NSCLC has been very limited so far [3].

Small RNA-based therapeutics offer exquisite advantages for therapeutic purposes in terms of flexibility, low toxicity and target-specificity, and permit us to reach non-druggable targets. Further, the introduction of several chemical modifications has been permitted to highly increase the RNA stability and improve their lack of immunogenicity [4,5,6]. Nevertheless, the efficacy is limited by: (1) the reduced RNA permeability limiting the crossing through the human body barriers; (2) the necessity to develop tissue-specific delivery strategies to allow drug accumulation in diseased sites avoiding the occurrence of off-target effects. Therefore, several delivery tools have been developed to improve barrier penetrance and intracellular uptake, including nanoparticles (NPs). NPs offer interesting and versatile carriers [7], but they generally show a preferential accumulation into the liver and require decoration with targeting ligands for a cell-specific delivery. To address cell-specific targeting, natural or artificial ligands have been explored. Among natural carriers, widely used is the *N*-acetyl-galactosamine (GalNAc) ligand recognized by the asialoglycoprotein receptor (ASGP) that is present on the hepatocyte surface and allows liver targeting [8]. Other natural ligands used as cell-specific carriers comprise transferrin [9] and small molecules, such as folate [10,11], employed for the targeting of the brain and other cancers (including NSCLC), respectively. In addition, artificial ligands, such as antibodies, proteins, peptides and aptamers, able to recognize cell surface receptors and permit cell-specific internalization, have been used for the targeted delivery. Among them, aptamers hold many useful features including good tumor penetration, cost-effectiveness, no toxicity, no immunogenicity and easy modification to improve their pharmacokinetic profile [12].

In this review, we provide an overview of recent advances of small RNA-based therapies for NSCLC. We will discuss key examples of the different small RNA subclasses highlighting their main features, formulations and the achieved results.

### 1.1. Therapeutic RNA Stability and Immunogenicity

A major hurdle for the therapeutic use of RNAs is represented by their instability due to the susceptibility to enzymatic degradation. To overcome this limitation, different chemical modifications to enhance RNA resistance to nucleases have been proposed. Substitutions at the 2′-position of the ribose have been frequently employed to address this issue. These include the introduction of 2′-fluoro, 2′-amino or 2′-O-metyl groups or the use of locked nucleic acids (LNAs) that contain a methylene bridge between the 2′-O to the 4′-C of the sugar. Modifications of the phosphate group, as the inclusion of phosphorothioates, or the capping at the 3′-terminus have been applied to improve RNA resistance to degradation, as well [13]. In addition to the improvement of the resistance, the use of alternative bases (e.g., pseu-douridines) has been found to ameliorate the stability of the duplex structures, enhanc-ing the functional activity of dsRNA molecules [14].

Importantly, chemical modifications of RNAs also improve their immunogenic profile. Different pathways, including Toll-like receptor (TLR) 3, 7 and 8, dsR-NA-dependent protein kinase (PKR), retinoid-acid-inducible gene I (RIG-I) and/or melanoma differentiation-associated gene 5 (MDA-5) pathways, physiologically recognize RNA molecules inducing innate immune response with the production of interferon (IFN) and pro-inflammatory cytokines. Small synthetic RNAs can be recognized by these systems and activate innate immunity [15], but this can be easily prevented through the design of non-immunogenic molecules avoiding the presence of immune-stimulatory motifs [16,17] and introducing modified nucleotides [18]. Concerning NSCLC, toxicology studies in rodents and primates have been performed for different small RNAs that are in clinical trials, revealing no controllable immune response activation [19,20,21]. However, the majority of therapeutically relevant RNA molecules proposed for NSCLC are still at an early preclinical phase of investigation and there are currently data on their effects on the host immune system in immunocompetent models available. Regarding the immunostimulatory properties of some small RNAs (mainly siRNAs and miRNAs), it should be considered that in some specific circumstances, the activation of the immune system represents a remedy for therapy, and the design of new RNA-based agents stimulating the host immune system has been explored for cancer and viral infection treatment [22].

### 1.2. ASOs

**Figure 2:**
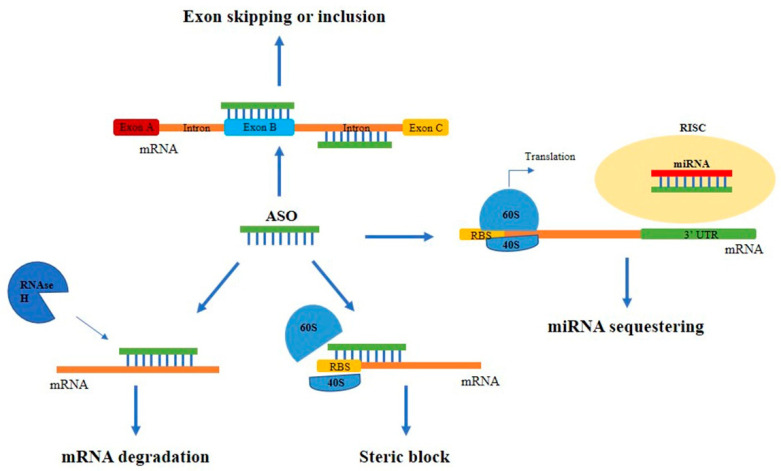
Main mechanisms of action of ASOs. Schematic representation of the different mechanisms of action of ASOs. ASOs can block protein expression by binding to the mRNA and leading to: (i) the RNase H-mediated mRNA degradation; (ii) the steric block of the correct ribosomal assembly; (iii) the alteration of the normal splicing causing exon skipping or inclusion. Alternatively, they can sequestrate miRNAs avoiding their binding to target mRNAs.

Antisense oligonucleotide-based therapy is a captivating approach to cancer treatment. ASOs are small (18-20 nucleotides) single-stranded modified oligonucleotides capable of hybridizing specific target mRNAs through base pairing recognition. The main mechanism of action of ASOs is the formation of hetero-duplexes with the mRNA in the cytoplasm, leading to the activation of the ubiquitous endonuclease RNase H and the hydrolysis of the complexes or to the alteration of the correct ribosomal assembly. Alternatively, ASOs can act by entering the nucleus and regulating mRNA maturation through the inhibition of the 5′ cap formation, of the normal splicing, or through the activation of the RNase H [23,24,25,26] (Figure 2).

ASOs activating RNase H have generally a specific composition with a central region of minimum of five DNA nucleotides called ‘gapmer’, flanked by RNA sequences, which increase the binding to the target. Steric block ASOs, instead, do not contain the DNA strand core, but include mixed modified nucleotides, such as LNAs, morpholinos, peptide nucleic acids and thiophosphoroamidates [27].

Antisense strategies have been used for decades with the purpose of downregulating over-expressed genes, but more recently have been employed even in the opposite situation, where the upregulation of therapeutic proteins could lead to a beneficial effect. This new approach is allowed by the development of new ASOs targeting miRNAs (called anti-miRNAs) that avoid miR binding to target mRNAs with a consequent upregulation of the translation. In addition, ASOs could be designed to target the 5′ untranslated region (UTR) of the mRNAs, which is often responsible for translational repression mainly through the formation of stem-loop structures [28].

Currently, ASOs are among the most advanced small RNA therapeutics in clinical trials, with several molecules approved by the Food and Drug Administration (FDA) for the treatment of different disorders. Among approved ASOs, there are: Fomivirsen for cytomegalovirus retinitis; Mipomersen for homozygous familial hypercholesterolemia; Eteplirsen and Golodirsen for Duchenne muscular dystrophy; Nusinersen for spinal muscular atrophy; Inotersen and Patisiran for polyneuropathy caused by hereditary transthyretin-mediated amyloidosis; Givosiran for acute hepatic porphyria; and Milasen, for neuronal ceroid lipofuscinosis 7 [29].

Although different drawbacks related to the safety and the selective and efficient delivery of ASOs still exist, they represent highly promising anti-cancer drugs.

Here, we present some examples of potential targets for antisense therapy in NSCLC (Table 1), summarizing the most relevant clinical data available so far.

ASOs have been widely applied to NSCLC therapy by targeting key genes regulators of the malignant phenotype, such as B-cell lymphoma 2 (Bcl2), Protein Kinase B (PKB/Akt-1), Kirsten Rat Sarcoma virus (KRAS), Vascular Endothelial Growth Factor (VEGF), Signal Transducer and Activator of Transcription 3 (STAT3), clusterin and Protein Kinase C alpha (PKC alpha).

KRAS is one of the most mutated genes in different human cancers, including NSCLC [35]. It is considered a good target for anti-cancer therapies and many KRAS inhibitors are currently under evaluation in clinical trials [36]. AZD4785 is an ASO containing 2′-4′ constrained ethyl residues (cEt). It targets mutated *KRAS* mRNA with high affinity and is able to induce its degradation. This ASO blocks pathways activated by KRAS hampering the proliferation of KRAS-mutated cancer cells [37]. Ross SJ et al. tested the efficacy of AZD4785 in multiple *xenograft* mouse models of NSCLC, demonstrating a good anti-tumor effect upon its systemic injection. In addition, they demonstrated ASO safety in primates, paving the way for the experimentation of its efficacy in a phase 1 clinical trial [37]. More recently, Wang et al. [38] reported a novel anti-KRAS ASO with improved antisense activity and pharmacokinetics. They designed a bottlebrush-like complex consisting of a DNA backbone, poly(ethylene glycol) (PEG) side chains and ASO overhanging. In this construct, PEG protects the ASO from enzymatic degradation, while ASO overhangs increase the chance of hybridization with the target. By using NSCLC cell models, authors showed that this molecule allows a higher antisense efficacy than unassembled hairpins and displays an improved retention time in vivo [38].

The over-expression of Bcl2 and its activator Akt-1 is responsible for the increased cancer cell viability, growth and apoptotic evasion in NSCLC [39,40]. Specific gapmer-based ASOs, called G3139 and RX-020, have been devised against *Bcl2* and *Akt-1* mRNAs, respectively, and tested in pre-clinical and clinical studies for NSCLC therapy. However, they did not reach success in clinical trials due to improper delivery. In order to improve ASO effectiveness, G3139 and RX-020 have been modified at the 5′ and the 3′ ends with 2′-O-methyl groups, and loaded in lipid NPs [30]. ASO-NP complexes demonstrated excellent cellular uptake and colloidal stability that let them gain a better anti-tumor effect in *xenograft* mouse models. Moreover, G3139 has been also incorporated in DOTAP/egg PC/cholesterol/Tween 80 lipid NPs and tested both in vitro and in vivo in NSCLC models. This complex gives an effective reduction in Bcl2 protein level and cell growth inhibition [41].

In order to target STAT3, a key transcription factor over-expressed in NSCLC, an ethyl-modified ASO, called AZD9150, has been designed by Hong D et al. and tested in vitro in multiple cell lines [19]. The ASO was 10–20 times more potent than the earlier generation (Gen 2.0) STAT3 ASOs, showing half-maximal inhibitory concentration (IC50) values for the most sensitive cells in the nanomolar range. Next, AZD9150 gave a strong inhibition of STAT3 in human primary patient-derived *xenograft* (PDX) models of NSCLC, colorectal cancer and lymphoma. Most importantly, the authors described a phase I dose-escalation study where AZD9150 antitumor activity as a single agent was observed in patients with highly treatment-refractory lymphomas and NSCLC. Currently, AZD9150 is being studied in a phase I/II trial alone or in combination with other anti-cancer drugs in patients with NSCLC, lymphoma and different advanced solid tumors [42,43]. In addition, again with the aim of targeting STAT3 in lung cancer, Njatcha and colleagues employed a cyclic 15 nucleotide length decoy called CS3D [34]. The circular oligonucleotide was obtained through the inclusion of two hexaethyleneglycol spacers that provide flexibility and thermal stability. Effects obtained upon decoy ASO transfection in 201T and H1975 lung cancer cells include the reduction in cell growth and proliferation and the increase in apoptosis.

Another key protein regulating important pro-oncogenic pathways in cancer, which can be therefore considered an interesting therapeutic target, is PKC. A 20-mer phosphorothioate ASO (ISIS 3521) has been synthesized to bind the 3′ UTR region of *PKC alpha* mRNA. ISIS 3521 has been tested alone or in combination with chemotherapeutics for the treatment of NSCLC and different refractory cancers [44]. The ASO entered phase III clinical trials in combination with carboplatin and paclitaxel, or with gemcitabine and cisplatin [45].

Combined therapeutic regimens in clinical trials for NSCLC include the use of an anti-clusterin ASO. Clusterin is a chaperone heterodimeric glycoprotein upregulated in many cancers, including NSCLC, and involved in the clearance of cellular debris and apoptosis. A 2′-methoxyethyl-modified phosphorothioate ASO (OGX-011) inhibiting the *clusterin* gene has been used in different kinds of tumors and is currently in phase I/II trial in combination with cisplatin and gemcitabine, and in phase III in combination with docetaxel for the treatment of advanced NSCLC [46,47].

An additional interesting application of ASOs in NSCLC was described to counteract angiogenesis. Recent studies have shown that VEGFA is up-regulated in squamous cell carcinoma, due to the overexpression of the long non-coding (lnc) RNA LINC00173.v1, a RNA sponge sequestering miR-511-5p [33]. Importantly, ASOs inhibiting LINC00173.v1 were developed and tested in mice, alone or in combination with cisplatin. Lung cancer cells were inoculated in mice and one week later, the ASOs were injected intraperitoneally once weekly for 4 weeks. Results showed the ASO ability to effectively inhibit tumor growth, increase mice survival and improve the therapeutic sensitivity to cisplatin [33].

Self-renewing tumor-initiating cells (TICs) represent a small population within the tumor mass that strongly contribute to tumor progression, recurrence and drug resistance. In order to target this population, Lin J et al. designed innovative splicing-modulating steric hindrance antisense oligonucleotides (shAONs) targeting the glycine decarboxylase (*GLDC*) gene, whose protein is a metabolic enzyme overexpressed in TICs of NSCLC, and necessary for their maintenance [31]. The developed ASOs efficiently induced exon skipping with an IC_50_ of 3.5–7 nM, disrupting the *GLDC* open reading frame and resulting in the inhibition of cell proliferation and colony formation in A549 cells and NSCLC tumor spheres (TS32). Most importantly, the best shAON strongly inhibited tumor growth in mice *xenografts* of TS32.

Recently, the GTP-binding protein ADP-ribosylation factor (ARF)-like (ARL) 4C (*ARL4C*) has been explored as a potential therapeutic target for NSCLC treatment. An ASO targeting *ARL4C*, called ASO-1316, was synthesized and tested in NSCLC evaluating cell proliferation, migration and tumor sphere formation [32]. Obtained results demonstrated the ASO-1316 ability to suppress lung cancer cell proliferation and migration in vitro, regardless of KRAS or EGFR mutation.

Thus far, the number of papers on ASO therapeutics is growing clearly indicating this class of molecules as a valid candidate for the effective therapy of NSCLC, preferably in combined regimens with other drugs, such as chemotherapeutics.

### 1.3. MicroRNAs

MiRNAs are a group of endogenous short ncRNAs (18-25 nucleotides in length) that negatively regulate gene expression at a post-transcriptional level. They have attracted great attention as anticancer therapeutics given to their pivotal role in tumorigenesis [48]. Indeed, miRNAs are involved in the regulation of numerous metabolic and cellular processes, including cell proliferation, differentiation, migration and survival, and can act as oncosuppressors or oncogenes (oncomiRs) [49]. One main feature of miRNA function is their ability to target multiple pathways simultaneously, thus offering the possibility to improve the therapeutic efficacy of the treatment, decreasing the occurrence of drug resistance, a frequent event when only one pathway is targeted.

**Figure 3:**
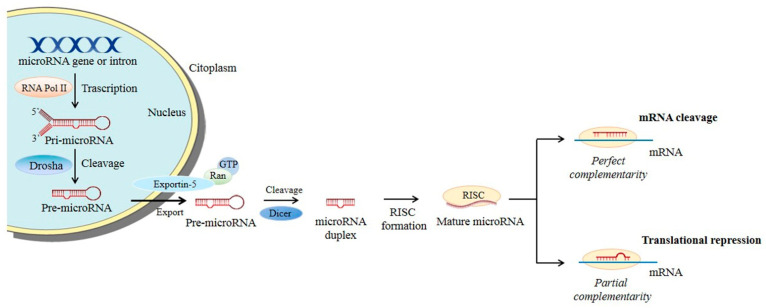
microRNA biogenesis. Main steps of microRNA processing. Briefly, miRNA transcripts (pri-miRNAs) are produced and cleaved by Drosha into pre-miRNAs. The pre-miRNAs are then exported into the cytoplasm by exportin-5–Ran-GTP and further processed into mature miRNA duplexes. The functional strand of the mature miRNA is thus guided by the RISC complex to the target mRNA permitting translational repression or mRNA cleavage.

MiRNA biogenesis (Figure 3) starts in the nucleus where the RNA polymerase II transcribes a pri-miRNA molecule, a double-stranded precursor with a polyadenylated stem-loop structure at the 3’ and a cap at the 5’. Two RNases III sequentially process this pri-miRNA: Drosha, which forms the pre-miRNA, that is then translocated into the cytoplasm, and Dicer, which processes the pre-miRNA into a mature miRNA. At this point, the non-functional strand (passenger) in the mature miRNA is degraded, while the functional one (guide strand) is loaded into a complex called RISC (RNA-induced silencing complex) and, based on its complementarity with the target mRNA, mediates mRNA degradation or translation inhibition.

Depending on the extent of miRNA expression in the tumor, different therapeutic approaches can be considered. When miRNAs are downregulated, they can be replaced by oligonucleotides that mimic their action or by viral vectors that allow miRNA expression to be restored. On the contrary, when miRNAs are over-expressed, they can be inhibited by: (i) synthetic anti-miRNAs, also known as antagomirs, that are ASOs complementary to the miRNA of interest able to induce the duplex formation and miRNA degradation; (ii) miRNA sponges that are competitive inhibitors containing multiple miRNA binding sites, allowing the simultaneous sequestering of multiple miRNAs [50]. The involvement of miRNAs in lung carcinogenesis has been widely documented by several studies focused on the differential expression pattern of miRNAs between lung tumor tissues and normal tissues [51].

Of note, despite different clinical trials for miRNA therapeutics are currently ongoing for a variety of cancers, including liver cancer, T-cell lymphoma, mesothelioma and lymphoma, only a few molecules are under clinical evaluation for lung cancer therapy, and only one for NSCLC [52].

The clinical study performed by van Zandwijk and colleagues involves the use of a miR-16 mimic enclosed in enveloped delivery vehicles (EDV), whose surface was functionalized with antibodies specifically targeting the epidermal growth factor receptor (EGFR), over-expressed in different cancer types, including NSCLC [53]. These complexes, called “TargomiR” showed encouraging results in a Phase I clinical trial in patients with recurrent malignant pleural mesothelioma and NSCLC.

In addition to this clinical study, a growing number of miRNA-based therapeutics are being tested in preclinical stages, providing some promising drug candidates for NSCLC treatment. Many studies addressed the stabilization and the selective and efficient delivery of these molecules, two key aspects for their successful clinical translation [54]. A brief description of the most studied miRNAs over-expressed or downregulated in NSCLC, is provided below and reported in Table 2.

Among over-expressed miRNAs, we want to mention miR-21, miR-150, miR-221/222, miR-96 and the miR-17/92a cluster.

*MiR-21* is commonly over-expressed in solid tumors and targets oncosuppressor genes, such as Phosphatase and TENsin homolog (*PTEN*), Programmed Cell Death 4 (*PDCD4*) and Tropomyosin 1 (*TPM1*), promoting cell growth, metastasis and escape from the apoptosis. The involvement of miR-21 in NSCLC tumorigenesis and drug resistance has been widely studied [55]. Therefore, its modulation by anti-miRNAs encapsulated in various delivery systems has been investigated and showed promising results. In this regard, an interesting study comes from Wang et al. [69], who employed a PEG-functionalized nanographene oxide (NGO-PEG) dendrimer to deliver the anti-miR-21 to NSCLC. In order to monitor the delivery efficacy, authors included an activatable luciferase reporter gene, containing three miR-21 complementary sequences in the 3′ UTR, so that miR-21 inhibition results in an increase in the luciferase activity. The complexes effectively delivered the anti-miR-21 into the cytoplasm upregulating luciferase intensity, restoring PTEN expression and inhibiting cell migration and invasion. Moreover, the intravenous administration of the complex in NSCLC mouse xenografts allowed efficient delivery of the anti-miRNA.

*MiR-150* has been proposed as a therapeutic target in different cancers, including NSCLC [56]. In particular, it has been reported to directly regulate the expression of Forkhead-box O 4 (*FOXO4*) and enhance tumor cell metastasis in NSCLC, both in vitro and in vivo in mouse *xenograft* models [70].

*MiR-221/222* has been found to be upregulated in TNF-related apoptosis-inducing ligand (TRAIL)-resistant NSCLC cells. Its inhibition has been demonstrated to improve TRAIL sensitivity by targeting cyclin-dependent kinase inhibitor 1B (*p27Kip1*) [57]. In addition to its therapeutic potential, miR-221 has been also proposed as a marker for early diagnosis and NSCLC screening [71].

*MiR-96* is a potent oncomiR, which increases NSCLC chemoresistance by reducing the expression of Sterile Alpha Motif Domain Containing 9 (*SAMD9*) [58]. It has been demonstrated that anti-miR-96, in combination with cisplatin, is able to upregulate *SAMD9* expression dramatically enhancing cisplatin-induced apoptosis. Although only in vitro data were provided, the study suggests a new approach to improve response to chemotherapy in NSCLC.

*MiR-17/92* cluster includes six family members: miR-17, miR-18a, miR-19a, miR-19b-1, miR-20a and miR-92a. Among them, miR-19 is the leading oncogenic player. The miR-17/92 cluster is typically amplified in lung cancer and targets different genes, such as Hypoxia-inducible factor 1-alpha (*HIF-1α*), *PTEN*, BCL2-like 11 (*BCL2L11*), cyclin dependent kinase inhibitor 2A (*CDKN2A*) and Thrombospondin-1 (TSP-1), which are involved in cancer cell death and proliferation [59]. Matsubara et al. found that ASO-inhibiting miR-17-5p and miR-20a induced apoptosis in cancer cells overexpressing miR-17-92 [72].

Furthermore, several studies described the deregulation of miR-19a in various cancer types, and demonstrated that its upregulation strongly correlates with increased metastasis, invasiveness, and a poor prognosis in NSCLC [73]. The study conducted by Jing Li and colleagues showed that the upregulation of miR-19 (miR-19a and miR-19b-1) in A549 and HCC827 lung cancer cells triggered EMT transition, which led to mesenchymal-like morphological changes and increased cell migration and invasion. The authors also demonstrated that the miR-19 target, PTEN, had an important role in these processes, which were reverted when the miRNA was silenced [60].

In addition to over-expressed miRNAs, many others are downregulated, acting as tumor-suppressor genes. Below, we discuss some of the most interesting tumor-suppressor miRNAs in NSCLC.

*MiR-Let-7* family includes 11 members, among which let-7a, let-7c, and let-7g are frequently downregulated in lung cancer patients. MiR-let-7 regulates the expression of several genes involved in cell proliferation and cell cycle, including cMyc, high-mobility group A (*HMGA*), STAT3, Janus-activated kinase 2 (*JAK2*), KRAS, Cyclin-Dependent Kinase 6 (*CDK6*), Homeobox A9 (*HOXA9*), transforming growth factor beta receptor 1 (*TGFBR1*), B-cell lymphoma-extra-large (*BCL-XL*) and Mitogen-activated protein kinase 3 (*MAP4K3*) [61,62,63,64]. It has been widely demonstrated that transfection of miR-Let-7 family members significantly decreased the proliferation rate of NSCLC cells and improved the sensitivity to chemo and radiotherapy [74,75,76]. Further, it has been reported that Let-7 reduces tumor formation in vivo, in NSCLC orthotopic models following intranasal administration, modulating *KRAS* expression. Notably, an aptamer-based strategy for the targeted delivery of miR-Let-7g has been developed by Esposito CL and colleagues and successfully applied to NSCLC both in vitro and in vivo (see the paragraph on Aptamers for more details) [77].

*MiR-29* family, including miR-29a, 29b and 29c, has been widely studied in NSCLC. A first report showed its ability to revert aberrant DNA methylation in lung cancer, by targeting the de novo DNA methyltransferases (DNMTs) 3A and 3B. This leads to the re-expression of methylation-silenced tumor-suppressor genes, such as fragile histidine triad protein (*FHIT*), *CDKN2A*, cadherin-13 (*CDH13*), cadherin-1 (*CDH1*) and Ras Association Domain Family Member (*RASSF1A*) both in vitro and in vivo [65]. Next, Wu et al. demonstrated that miR-29 transfection by cationic lipoplexes significantly reduced the expression of cell division protein kinase 6 (*CDK-6*), *DNMT3B* and myeloid leukemia cell differentiation protein 1 (*MCL1*) and inhibited tumor growth in subcutaneous *xenograft* models of A549 NSCLC cells [78]. More recently, Xing et al. confirmed the therapeutic potential of miR-29, by employing an *N*-isopropylacrylamide-modified PEI (namely PEN) as a carrier for miR-29a. The authors demonstrated the complex efficacy in allowing miR-29a delivery and processing with a consequent anti-proliferative and anti-migratory effect on A549 NSCLC cells [79].

*MiR-200* family is known as a negative regulator of the EMT due to its ability to target Zinc finger E-box-binding homeobox (*ZEB*) family members, acting as translational repressors that promote EMT [66]. MiR-200 decreases tumor survival by reducing the expression of *VEGF* and *VEGF-R1* [67]. In a mouse model of lung adenocarcinoma (KrasLSL-G12D/+; Trp53flox/flox), metastasis was significantly increased by the deletion of miR-200c/141, resulting in a desmoplastic tumor stroma [80]. Within the stroma, the NOTCH signaling pathway is promoted by the downregulation of miR-200 in cancer-associated fibroblasts, improving the ability of cancer cells to metastasize [80]. In a group of NSCLC patients with adenocarcinoma, Tejero and colleagues recently showed that high levels of miR-141 and miR-200c are associated with shorter overall survival [81]. In addition, Cortez and co-workers have shown that miR-200c promotes radiosensitivity of lung cancer cells by controlling the oxidative stress response through the direct regulation of Peroxiredoxin 2 (*PRDX2*), GA-binding protein (*GAPB/Nrf2*) and Sestrin 1 (*SESN1*), and by preventing the repair of radiation-induced double-strand breaks [82]. The therapeutic potential of miR-200c in combination with other treatments, such as radiation, was also demonstrated in vivo.

*MiR-34* family has been found to be deregulated in several human malignancies, and is considered a tumor-suppressive miRNA group due to its synergistic effect with the tumor suppressor gene *p53* [83,84]. The miR-34 family has been shown to inhibit the translation of genes involved in cell growth, proliferation, cell cycle control and anti-apoptotic signaling [85]. In addition, several preclinical studies have shown the big potential of miR-34a in cancer treatment [86]. Kasinski et al. showed that miR-34a is a valuable therapeutic option for NSCLC treatment. They concluded that the administration of miR-34a to lung cancer patients with KRAS -positive and p53-negative tumors, slows tumor growth and potentially increases survival [87]. Notably, miR-34a was the first miRNA restoration therapy investigated in a clinical trial. Although the trial was ultimately unsuccessful [68], miR-34a is expected to be included in further miRNA mimic studies due to its potent tumor-suppressive effect in a variety of tumor subtypes.

### 1.4. Small Interfering RNAs

Small interfering RNAs (siRNAs) are synthetic double-stranded RNA (dsRNA) molecules, with a length of 20-27 nucleotides, able to induce the silencing of a specific target gene. SiRNAs enter the processing machinery of miRNAs (Figure 3), being converted into mature duplexes by DICER and forming a complex with RISC that guides the antisense strand in recognizing the target mRNA, inducing its degradation [88,89,90].

SiRNA-based strategies show the advantage of permitting the silencing of any gene, even undruggable targets. Importantly, as for other small RNAs, the development of advanced systems, suitable for targeted and efficient delivery, represents a primary challenge for their clinical development. Since their discovery in 1998 [91], many progresses have been completed in their application for the treatment of many diseases [92], and different siRNAs, generally formulated in different delivery systems, have been approved or are currently in clinical trials. The FDA and the European Medicines Agency (EMA) have already approved some siRNA-based drugs for the treatment of various human pathologies, different from cancer. In 2018, the first siRNA therapeutic, named Patisiran, was approved for the treatment of hereditary transthyretin amyloidosis [93]. Then, Givosiran was approved by the FDA for the treatment of acute hepatic porphyria [94], Lumasiran for the treatment of type 1 Primary hyperoxaluria [95], and Inclisiran for the homozygous familiar hypercholesterolemia therapy [96].

Additional siRNA-based approaches are currently in clinical trials; some of them are under consideration for cancer treatment [97], even if these are in a less advanced stage than those applied for the treatment of other human pathologies.

Among them, a phase I clinical study is recruiting patients to investigate the safety and the efficacy of NBF006 for advanced therapy of solid tumors, including NSCLC. NBF006 is a novel lyophilized lipid NP formulation, which delivers a siRNA inhibiting the expression of glutathione-S-transferase P that is strongly up-regulated in many cancer types, especially in KRAS-driven tumors [98].

Furthermore, concerning NSCLC, multiple therapeutic targets for siRNA molecules have been identified and many preclinical studies are demonstrating the efficacy of siRNA-based approaches for the treatment of this pathology (Table 3) [99].

Many efforts have been devoted to overcoming NSCLC resistance to therapy, a feature that greatly limits patient outcome. Among the available therapeutic options, platinum-based chemotherapies (cisplatin and paclitaxel) are the standard of care for advanced NSCLC, while TKIs against the EGFR, gefitinib and erlotinib, are the first-line treatment for patients with EGFR mutation. However, despite the improvements achieved with such drugs, the development of mechanisms of resistance is very frequent [100]. To bypass the acquired drug resistance to TKIs, the use of siRNA-based approaches has been proposed by Chen et al. Authors demonstrated that co-administering TKIs with an anti-EGFR siRNA increases the apoptotic rate in different NSCLC lines [101]. Similarly, Garbuzenko et al. analyzed the efficacy of a pool of siRNAs targeting the four EGFR-TK forms in combination with paclitaxel [102]. The siRNAs were encapsulated in a unique nano-delivery system that, in order to achieve the specific targeting of NSCLC was decorated with a synthetic Luteinizing Hormone-Releasing Hormone (LHRH) decapeptide. This peptide recognizes the extracellular LHRH receptor over-expressed in lung cancer cells. The complexes demonstrated a good bio-distribution and an improved anti-cancer effect, compared to single treatments, both in vitro and in vivo in orthotopic NSCLC mouse models [102].

The possibility of enhancing erlotininb efficacy in NSCLC cells has been instead explored by Surresh et al. through the targeting of the AXL receptor [103]. Similarly to EGFR, AXL controls different pathways implicated in cancer cell proliferation, and has been correlated to the resistance to EGFR-targeting therapies [104]. The authors used an anti-AXL siRNA in H820 cells, and observed a reduction of about 80% in the expression levels of AXL, and a subsequent increase in p53 and the inactivation of the PI3K/Akt pathway. When erlotinib was co-administered with the siRNA, cell migration ability was inhibited, and apoptosis was increased [103].

**Table 3:** Main siRNA-based therapeutics applied to NSCLC therapy.

Target	Delivery System	Stage of Development	Ref.
*Glutathione-S-transferase P*	Lipid NP	Clinical (Phase I)	clinical trial #NCT03819387
*EGFR*	Cationic liposome	Preclinical (in vitro)	[101]
*EGFR*	LHRH-decorated-Nanostructured Lipid Carrier	Preclinical (in vitro + in vivo)	[102]
*AXL*	Antibody-conjugated gelatin NP	Preclinical (in vitro)	[103]
*KRAS*	Polimeric NP	Preclinical (in vitro)	[105]
*KRAS*	Gelatin NP	Preclinical (in vitro)	[106]
*KRAS (G12C mutation*)	Cationized Gelatin NP	Preclinical (in vitro)	[107]
*Mad2*	Chitosan NP	Preclinical (in vitro + in vivo)	[108]
*Survivin*	Hyaluronic acid nanosystem	Preclinical (in vitro + in vivo)	[109]
*Survivin*	Polimeric NP	Preclinical (in vitro + in vivo)	[110]
*Survivin*	Polimeric NP	Preclinical (in vitro)	[111]
*SOX2*	-	Preclinical (in vitro)	[112]
*VEGF*	(shRNA) Lentivirus	Preclinical (in vitro)	[113]
*VEGF*	Lipid-Calcium-Phosphate NP	Preclinical (in vitro + in vivo)	[114]
*VEGF*	Polimeric NP	Preclinical (in vitro + in vivo)	[115]

Of note, siRNA-based strategies in NSCLC have been applied to target *KRAS* oncogene [116]. Xue et al. [105] combined *KRAS* siRNAs and miR-34. The combined regimes resulted in reduced tumor growth and acquired sensitization to cisplatin in vitro and in vivo in *KRAS-p53* mutated mouse models of lung adenocarcinoma. Skirar et al. [106] have instead developed a tri-block nanoparticle (TBN) complex decorated with a conjugate containing a *KRAS* siRNA linked to the anti-EGFR antibody Cetuximab, and loaded with gefitinib. TBNs were tested in H23 cells demonstrating to efficiently reduce the expression levels of KRAS and restore gefitinib sensitization.

More recently, Sreedurgaalakshmi et al. [107] have described a similar approach. They developed a gelatin–antibody delivery system containing the anti-EGFR antibody Cetuximab linked to a siRNA specific for the *KRAS G12C* mutated form [117]. The authors demonstrated the ability of the complex to internalize in a receptor-mediated manner, efficiently silencing *KRAS G12C* and sensitizing cancer cells to gefitinib [107].

Moreover, in order to increase the NSCLC response to cisplatin, Nascimento et al. [108] used a siRNA targeting mitotic arrest deficient 2 (*Mad2*), an important mediator in DNA damage response. Authors demonstrated the therapeutic efficacy of siRNA both in vitro and in vivo, in subcutaneous *xenograft* mouse models. In other studies, the targeting of survivin, a protein involved in the regulation of cell proliferation and apoptosis, has been proposed to revert NSCLC cisplatin resistance [109,110,118]. In all these studies, it has been observed that the silencing of survivin can revert the resistance to cisplatin, both in vitro and in vivo. In addition, survivin was used as a target for siRNA-based therapies by Chen et al. [111]. The authors developed a new nano-delivery platform employing Poly γ-glutamic acid (γ-PGA) and PEG, and decorated it with anti-survivin siRNAs. In such a complex, the γ-PGA confers nontoxicity, biocompatibility and tumor specificity, being recognized by the γ-glutamyl transpeptidase over-expressed in many tumor types, while PEG shell enhances NP stability. These complexes demonstrated efficient survivin gene silencing and enhanced Doxorubicin-induced apoptosis in A549 NSCLC cells. Always regarding NSCLCs sensitivity to cisplatin, recently, Cheng and co-workers [111] analyzed the level of sex-determining region Y-box 2 (*SOX2*) in 45 NSCLC patients’ biopsies and found a direct correlation between *SOX2* levels and patients’ outcomes. Importantly, the authors demonstrated that when cells were treated with a *SOX2* siRNA, cisplatin sensitivity was restored.

Further, another appealing therapeutic target in NSCLC, worthy of mentioning, is the VEGF. The over-expression of *VEGF* is very common in NSCLC and negatively correlates with the overall NSCLC patients’ survival [119]. The targeting of *VEGF* offers the possibility to inhibit tumor angiogenesis, a key process in cancer progression and metastasis. The inhibition of *VEGF* through siRNA-based strategies has been widely explored in NSCLC systems. In one of the first studies, Feng et al. [113] used a lentivirus-mediated shRNA (short hairpin RNA) technology to silence *VEGF-C* in A549 cells. They demonstrated that *VEGF* silencing suppressed tumor cell growth, migration and invasion in vitro, and tumor growth and angiogenesis in vivo in lung cancer *xenograft* models, upon lentivirus tail vein injection. Yang and co-workers [114] obtained similar results. They used a pool of siRNAs targeting *VEGF*, Human double minute 2 protein (*HDM2*) and *c-myc*, and applied a Calcium–Phosphate (CaP)-based NP system, named the Lipid/Calcium/Phosphate (LCP), for their delivery. The developed LCPs contained a CaP core stabilized with DOPA, coated with cationic lipids and linked to anisamide ligand for the specific targeting of tumor cells expressing the sigma receptor. By such a strategy, the authors observed an efficient siRNA delivery to sigma receptor-positive cells with a reduction in the expression of target genes, both in vitro and in vivo in mouse models. Specifically, they found reduced tumor growth and absence of toxicity in vivo [114]. The next studies analyzed the efficacy of *VEGF* silencing in combination with chemotherapeutics. For example, Zhang’s group [115] investigated the co-delivery of an anti-*VEGF* siRNA with etoposide (ETO). Authors developed multi-functional NPs consisting of PEGylated histidine-grafted chitosan-lipoic acid (PHCL) polymer used to coat liposomes loaded with *VEGF* siRNA and ETO. The developed complexes showed improved tumor penetration and cellular internalization, good silencing and anti-proliferative effects in vitro. Most importantly, in orthotopic mouse models of lung cancer, NPs allowed a more effective inhibition of tumor growth and metastasis, as compared to single therapies [115].

The reported examples want to provide an overview of the therapeutic potential of siRNA-based drugs for NSCLC therapy. It should be noted that more studies continuously appear in the literature proposing new promising siRNA targets and delivery strategies.

### 1.5. Aptamers

Aptamers are synthetic single-stranded nucleic acids produced through a combinatorial chemistry technique, named SELEX (Systematic Evolution of Ligands by EXponential Enrichment) [120,121], useful as highly specific targeting agents for different biomedical applications. Their mechanism of action is similar to that of monoclonal antibodies. In fact, by folding into particular three-dimensional shapes, aptamers bind with high affinity and specificity their targets, the reason why they are also called “chemical antibodies” (Figure 1) [122]. Compared to mAbs, aptamers show a faster, cheaper and easier production, a higher batch fidelity, no immunogenicity and greater plasticity, in the sense that they can receive various chemical modifications to improve their pharmacodynamic and pharmacokinetic properties. Similarly to mAbs, aptamers often show inhibitory activity on their target molecule, as they compete with the binding of endogenous ligands. This feature makes aptamers potential therapeutics for cancer [123]. Moreover, aptamers against cell surface receptors may undergo intracellular receptor-mediated uptake, representing attractive molecules for targeted delivery [124,125].

Many inhibitory aptamers have been developed for the treatment of different human pathologies, including cancer. Among RNA aptamers proposed for NSCLC therapy, we can mention some that gave good results in preclinical evaluations (summarized in Table 4).

The CL4 aptamer, directed against the human EGFR, was selected by differential cell-SELEX for NSCLC and characterized by Esposito et al. [126]. The aptamer was proven to bind with high affinity the extracellular domain of the human EGFR, with a calculated Kd of 10 nM. The authors demonstrated aptamer specificity for EGFR-expressing cells and the capacity to inhibit EGFR activation in vitro competing with the binding of the endogenous ligand. Because of this inhibition, the CL4 aptamer significantly inhibited cell viability and induced apoptosis in vitro. Moreover, it demonstrated good antitumor efficacy in vivo in NSCLC mouse *xenografts*, efficiently inhibiting tumor growth with a significant reduction (~60%) of the tumor mass. Very interestingly, the CL4 aptamer induced apoptosis also in cells resistant to gefitinib and cetuximab, the most commonly used EGFR-inhibitors. This indicates CL4 as a potential alternative to the currently used EGFR-inhibitors for NSCLC therapy [126].

Further, the same group selected and characterized another RNA aptamer useful for NSCLC treatment [127]. This is the GL21.T aptamer, binding the extracellular domain of the human tyrosine kinase receptor AXL with a calculated Kd of 13 nM. The authors demonstrated aptamer specificity for the AXL receptor, also respected other members of the same family, and the capacity to inhibit its phosphorylation following Gas6 ligand stimulation.

As a consequence of this inhibition, the GL21.T aptamer showed only a poor inhibitory capacity of cell viability in vitro (~20%), and no sensitization towards the most commonly used chemotherapeutics (cisplatin, paclitaxel and TRAIL). It instead demonstrated a strong inhibition of cancer cell migration induced by unspecific (FBS) or specific (Gas6) stimuli and a significant reduction in colony formation in semisolid media. Most importantly, the aptamer showed high antitumor efficacy in vivo in NSCLC mouse *xenografts*, leading to a significant (~70%) reduction in the tumor masses [127].

Another RNA aptamer, RA16, demonstrating an antitumor activity in NSCLC, was isolated through in vivo SELEX by Wang et al. [128]. The chemically synthesized version of this aptamer (syn-RA16) showed a high affinity (24.75 ± 2.28 nM) and a specific binding to H460 NSCLC cells. Despite the fact its molecular target has not been identified, the aptamer demonstrated an anti-proliferative effect in vitro, indicating a potential inhibitory effect on some receptors expressed on the surface of NSCLC cells. The authors also described aptamer biodistribution in vivo in NSCLC mouse *xenografts*, observing a good accumulation in tumor masses by 30 min to 4 h following injection, with a peak at 2 h. Further, a truncated version of the RA16 aptamer (S3) resulted to preserve the binding capacity on H460 cells (Kd of 63.20 ± 0.91 nM), and showed anti-proliferative activity in vitro, even if to a lower degree if compared with the long version of the aptamer [128].

**Table 4:** Main examples of aptamer and aptamer complexes applied to NSCLC therapy.

Aptamer	Target	Conjugation	Functional Secondary Molecule	Therapeutic Effect	Ref.
CL4	EGFR	-	-	Cell viability inhibition, tumor mass reduction in vivo	[126]
		aptamer decorating DSPE-PEG2000 drug-loaded nanomicelles	Salinomycin	Cell viability inhibition, tumor growth inhibition in vivo	[129]
		aptamer decorating siRNA-loaded exosomes	Survivin siRNA	Tumor growth reduction in vivo	[130]
GL21.T	AXL	-	-	Cell migration inhibition, tumor mass reduction in vivo	[127]
		Covalent complexes	miR-Let-7g	Tumor growth inhibition	[77]
		Covalent complexes	miR-212	TRAIL oncosuppressor pathway restoration	[131]
		Stick-based complexes	miR-34c	Erlotinib sensitivity restoration	[132]
		Stick-based non-covalent complexes	miR-137	Cell viability and migration inhibition	[133]
RA16	-	-	-	Cell viability inhibition	[128]
		Drug intercalation	Epirubicin	Antitumoral activity in vivo	[128]
Anti-CD133 aptamer	CD133	Aptamer decorating drug-loaded liposomes	Docetaxel	Cell proliferation inhibition and antitumour activity in vivo	[134]
Anti-CD133 + Anti-CD44 aptamers	CD133 and CD44	Aptamers decorating drug-loaded nanomicelles	Gefitinib	Cell proliferation and tumorsphere formation inhibition	[135]

Interestingly, aptamers, in addition to being antagonists of receptors over-expressed in cancer, following binding to their specific target on the cell surface, can internalize in a target-mediated manner. Therefore, they can be used as carriers for the selective delivery of therapeutics in cancer cells, decreasing the side effects related to non-specific drug distribution in healthy tissues [136]. Notably, if the aptamer used for the delivery of an anti-cancer therapeutic also exerts an antitumor activity, the therapeutic efficacy of the aptamer-based complex is improved, resulting in a multifunctional tool.

**Figure 4:**
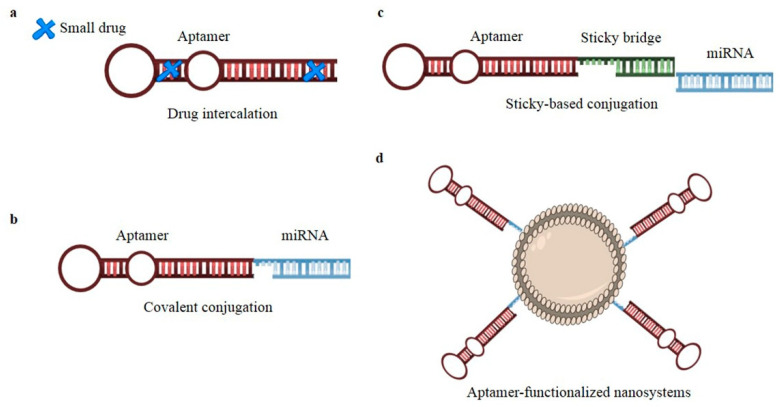
Schematic representation of aptamer-based complexes applied to NSCLC therapy. Aptamer complexes developed for the targeting of NSCLC were generated by: (**a**) direct drug intercalation within the aptamer structure; (**b**) aptamer covalent conjugation to miRNAs in which the aptamer sequence is extended with one of the miRNA strand and then annealed with the other miRNA strand; (**c**) aptamer non-covalent conjugation to miRNAs through a stick-based strategy in which the aptamer and miRNA passenger strand are extended with complementary stick sequences to allow their annealing; (**d**) nanosystem decoration with a targeting aptamer.

Among aptamer-based complexes applied to NSCLC treatment (schematized in Figure 4), an interesting example is the RA16-epirubicin molecule [128], in which epirubicin was non-covalently intercalated into a PEGylated version of the RA16 aptamer (Figure 4a), generating a bi-functional molecule with increased serum stability and half-life and an improved antitumor activity both in vitro and in vivo, in H460 *xenograft* mouse models.

Further, some aptamers have been used for the targeted delivery of small RNA therapeutics to NSCLC. In this regard, the therapeutically active GL21.T aptamer [127] was developed for the targeted delivery of therapeutic miR-let-7g, generating a multifunctional complex in which the aptamer was covalently linked to the miRNA [77] (Figure 4b). The aptamer-miRNA conjugate was demonstrated to be efficiently internalized in AXL-expressing cells, allowing correct miRNA processing and target gene silencing. Further, the multifunctional complex demonstrated an improved efficacy in vivo in NSCLC *xenograft* models, significantly inhibiting tumor growth [77].

Using a similar conjugation strategy, the same aptamer was used by Iaboni and co-workers for the targeted delivery of miR-212, a tumor-suppressor miRNA in NSCLC [131]. Interestingly, the authors compared the internalization rate of the miR-212 induced by the aptamer with that induced by a liposome-based transfecting agent. Despite a less miRNA internalization rate induced by the aptamer, a similar downregulation of *PED*, target of miR-212 and restoration of TRAIL oncosuppressor pathway was observed in the two different systems [131].

By using a different conjugation strategy, named “stick-based” approach (Figure 4c), the GL21.T aptamer was also developed for the targeted delivery of miR-34c-3p [132] and miR-137 [133] in NSCLC. The “stick-based” approach provides the use of brief complementary “stick” sequences to allow the annealing of the aptamer with the passenger strand of the miRNA.

In the study by Russo et al. [132], the authors showed an efficient aptamer-mediated internalization of the miR-34c in AXL-expressing cells and a restoration of cell sensitivity to Erlotinib in vitro.

In the work by Nuzzo et al. [133], the aptamer-miR-137 conjugate was revealed to significantly inhibit cell viability and migration both in vitro and in ex vivo systems of NSCLC, and tumor growth in *xenograft* NSCLC models.

Some examples of multivalent NP-based compounds decorated with aptamers (Figure 4d) as specific targeting agents have been also described in the literature [137]. Among them, there are different studies exploring the employment of anti-EGFR aptamers to this scope. Zhang et al. [138] encapsulated the HHT (homoharringtonine), a natural alkaloid with potent anti-cancer effects, in PLGA (poly(lactic-co-glycolic acid)) nanoparticles decorated with an anti-EGFR RNA aptamer. If compared to free HHT, the nano-complex showed an improved anti-cancer activity in vivo in subcutaneous tumor *xenograft* mouse models [138].

Leng et al. [129] used a thiolated version of the CL4 RNA aptamer [126] targeting EGFR to decorate DSPE-PEG2000 nanomicelles loaded with salinomycin, an antibacterial and coccidiostat ionophore drug which showed potent therapeutic activity against cancer stem cells (CSCs) in various tumors [139]. DSPE-PEG2000 nanomicelles were chosen for their small size which can allow an increased penetration. However, the authors demonstrated that nanomicelle decoration with the anti-EGFR aptamer leads to an increase in drug permeability rate. The nano-complex successfully inhibited cell viability in vitro and tumor growth in vivo in *xenografts* of NSCLC [129].

In addition to NPs, recently, exosomes are revealing as ideal multidrug vehicles for cancer therapy [140]. Exosomes are small cellular vesicles (50–150 nm diameter) with the capability of naturally fusing with the plasma membrane. Li and colleagues [130] generated a CL4 aptamer [126] decorated exosome system loaded with anti-survivin siRNAs for NSCLC therapy. Authors proved that the aptamer conferred to the exosomes an enhanced ability to bind and enter NSCLC cells. Efficient surviving gene silencing, sensitization to cisplatin treatment and inhibition of cancer cell growth were also demonstrated in vitro. Interestingly, an increased apoptosis rate was observed co-administrating the exosome complexes with the cisplatin. Moreover, the complexes effectively reduced tumor growth in vivo, in *xenograft* NSCLC models [130].

Another example of aptamer decorated nano-system for NSCLC treatment was developed by Ma et al. [134]. The authors generated liposomes loaded with Docetaxel and decorated with an anti-CD133 RNA aptamer targeting lung CSCs. Liposomes showed an increased cellular uptake when functionalized with the aptamer both in vitro and in vivo. In another study [135], the anti-CD133 aptamer was used together with the anti-CD44 aptamer to decorate nanomicelles loaded with gefitinib, for the selective targeting of different populations of lung cancer-initiating cells. In vitro results demonstrated the specific targeting of CD133^+^ and CD44^+^ cell populations, and the reduction in cancer-initiating cell proliferation and tumorsphere formation.

The reported examples demonstrated the relevance of aptamers in the field of RNA therapeutics for cancer treatment, as both antagonistic drugs and targeting moieties in multivalent complexes for improved non-toxic NSCLC therapy.

## 2. Conclusions

The studies described in this review highlight the great potential of small RNAs as therapeutics for cancer, with a specific focus on NSCLC, the most common cancer worldwide. Despite advances, resistance to conventional therapies, relapses and poor prognosis are very frequent. Innovative anti-cancer strategies, including nanosecond microwave pulses, have been applied to inhibit other types of cancer [141] and can be potentially used for NSCLC, even if no data on this kind of application are available so far.

Most importantly, growing numbers of important results are paving the way for the rational design of small RNA-based drugs as tools for a precision and personalized therapy of NSCLC.

However, despite advances, the broad clinical applicability of small RNAs therapeutics especially for cancer treatment has not been realized. Indeed, there are still hurdles to address, mainly related to their efficacy and targeted delivery. These aspects are greatly influenced by the trafficking mechanisms of the delivery systems and the necessity to target tissues different from the liver. Even though the mechanism of small RNA processing within the cells is not yet completely understood, it has been shown that upon cell internalization, only the 1% of RNAs escape from the endosomes for cytoplasmic delivery. This important limiting factor needs to be addressed for small RNA widespread clinical application. Even if many efforts have been devoted to finding strategies to improve endosome escape [142], a winning approach is still lacking and studies aimed to improve our understanding of trafficking mechanisms are required. Similarly, concerning targeted delivery, many targeting moieties have been proposed [143], but there are no systematic studies comparing different systems and showing how they influence drug efficacy and tolerability.

Another important consideration is that manufacturing issues need to be accomplished to realize the RNA therapeutic potential. Indeed, ideal clinical systems need to be synthesized with scalable chemistry, to show low batch variability and to have no toxicity at the effective dose and a good on-target/off-target ratio. In addition, they must permit redosing maintaining the same efficacy and safety.

All these elements are the main challenges to solve in the next future to accelerate the clinical development of small RNA therapeutics and produce extraordinary medical advances in NSCLC treatment.

## Figures and Tables

**Table 1:** ASO applications in NSCLC.

Name	Target	Phase of Development	Ref.
Aprinocarsen (ISIS 3521)	*PKC alpha*	Phase III with carboplatin and paclitaxel Phase III with gemcitabine and cisplatin	clinical trials #NCT00017407, #NCT00034268
Custirsen (OGX-011)	*Clusterin*	Phase III with docetaxel Phase I/II with gemcitabine/platinum-based regimen	clinical trial #NCT01630733, #NCT00138658
AZD4785	*KRAS*	Phase I	clinical trial #NCT03101839
AZD9150	*STAT3*	Phase I with chemotherapy	clinical trial #NCT03421353
Oblimersen (G3139)	*BCL2*	Phase II/III with docetaxel Phase I/II with paclitaxel Phase I with chemotherapy	clinical trial #NCT00030641, #NCT00005032, #NCT00017251
RX-020	*PKB/AKT1*	Preclinical	[30]
shAONs	*GLDC*	Preclinical	[31]
ASO 1316	*ARL4C*	Preclinical	[32]
LINC00173.v1 ASO	*LINC00173.v1*	Preclinical	[33]
CS3D	*STAT3*	Preclinical	[34]

**Table 2:** Most studied miRNAs over-expressed or downregulated in NSCLC.

miRNA	Function	Experimentally Validated Targets	Function	Ref.
Up-regulated miRNAs				
miR-21	Oncogene	PTEN, PDCD4, TMP1, SMAD 7	Promotes cell proliferation, metastasis and discourages apoptosis	[55]
miR-150	Oncogene	FOXO4	Associated with metastatic malignant lung cells and tissues	[56]
miR-221/222	Oncogene	P27kip1, TIMP3, PTEN, PUMA	Promotes TRAIL resistance	[57]
miR-96	Oncogene	SAMD9	Promotes cisplatin chemoresistance	[58]
miR-17-92	Oncogene	PTEN, CDKN2A, BCL2L11, PPP2R5, TSP-1, HIF-1α	Promotes proliferation, metastasis and invasion, linked with short survival	[59,60]
Down-regulated miRNAs				
miR-Let-7 family	Tumor-suppressor	KRAS, c-MYC, CDK6, TGFBR1, BCL-XL, STAT3, JAK2, MAP4K3	Suppresses tumor growth and invasion	[61,62,63,64]
miR-29 family	Tumor-suppressor	DNMT3A and 3B, FHIT, CDKN2A, RASSF1A, MCL1	Epigenetic regulation of gene expression	[65]
miR-200 family	Tumor-suppressor	ZEB1, VEGF, VEGFR1 PRDX2, GAPB/Nrf2, SESN1	Suppresses angiogenesis, epithelial–mesenchymal transition (EMT) and promotes radiosensitivity	[66,67]
miR-34	Tumor-suppressor	PD-L1, MET, MYC, PDGFR-α, CDK4/6, BCL2;	Inhibits proliferation and induces apoptosis	[68]

## Data Availability

Data sharing not applicable.
